# Acceptance of Pulmonary Telerehabilitation Among Healthcare Practitioners

**DOI:** 10.7759/cureus.87593

**Published:** 2025-07-09

**Authors:** Gayathri Pandurangam, Swathi Gurajala, Laila Almarhoon, Leenah Hassan, Talah Aljaloud, Rahaf Aljadani, Walaa Alshahrani, Shoug Y Al Humoud, Sally Yusef Abdulrahman Abed

**Affiliations:** 1 Department of Respiratory Care, College of Applied Medical Sciences in Jubail, Imam Abdulrahman Bin Faisal University, Dammam, SAU

**Keywords:** behavioral intention (bi), health care practitioners, pulmonary rehabilitation, technology acceptance model., tele-rehabilitation

## Abstract

Introduction: Pulmonary rehabilitation is a clinical approach designed to support individuals with chronic respiratory diseases through structured exercise and educational strategies. Pulmonary telerehabilitation (PTR) represents a remote adaptation of this approach, offering services through virtual platforms. This model may enhance patient engagement and adherence due to its convenience and accessibility. However, its implementation remains inconsistent across healthcare institutions, potentially limiting its benefits for both healthcare providers and patients.

Objective: This study aimed to assess the level of acceptance and identify the factors and barriers influencing the intention of healthcare practitioners (HCPs) to use PTR in Saudi Arabia.

Method: A cross-sectional survey was conducted among HCPs involved in pulmonary rehabilitation across Saudi Arabia between August 2024 and May 2025. Participants completed an online questionnaire using the pre-validated Tele Pulmonary Rehabilitation Acceptance Scale (TPRAS), which measures behavioral intention (BI) as the dependent variable, and perceived usefulness (PU) and perceived ease of use (PEOU) as independent variables. Linear regression analysis was applied to examine the impact of PU and PEOU on BI.

Results: A total of 59 practitioners participated in the study. Among them, 82.77% demonstrated a strong willingness to engage with PTR. PU was identified as the most influential factor shaping behavioral intention. Of the HCPs, 76.27% accepted the PEOU of PTR equipment, while 81.35% of HCPs had positive BI to use PTR. Commonly cited challenges included unstable internet connectivity and limited patient engagement.

Conclusion: The intention among HCPs to implement PTR appears to be strongly associated with their perceptions of its usefulness and ease of application. Addressing identified barriers could further support the adoption of this technology in clinical practice.

## Introduction

The global prevalence of chronic respiratory diseases, the fourth leading cause of death worldwide, reached 3.5 million deaths in 2021, accounting for 5% of global deaths and the eighth leading cause of poor health worldwide. Chronic obstructive pulmonary disease (COPD) is the most common chronic respiratory disease with a considerable financial burden due to limited workplace and home productivity and the cost of medical treatment, followed by asthma and interstitial lung disease [1].

For people with long-term respiratory disorders, pulmonary rehabilitation is a comprehensive, lifelong intervention. It involves detailed patient evaluation and personalized plans, including exercise training, educational support, and behavioral education. These interventions aim to enhance physical health, increase exercise capacity, improve daily life activities, and boost overall stamina. Education about disease management empowers patients to take control of their health and make informed decisions.

However, pulmonary rehabilitation has limitations. Many patients face physical barriers that hinder participation, and transportation is a significant issue for those in rural areas [2]. Telehealth, a branch of e-health, addresses these challenges by using telecommunications to deliver healthcare, information, and education remotely [3]. Pulmonary telerehabilitation (PTR) offers services such as patient education, exercise training, and monitoring for chronic lung disease patients [4]. PTR is particularly beneficial for those with mobility issues, those living far from clinics, or those concerned about infection exposure. It can reduce treatment costs and travel time and improve physical activity, self-monitoring, and quality of life [5]. Patients have shown high satisfaction and compliance with PTR [6]. However, technical challenges due to inadequate skills among healthcare practitioners (HCPs) and patients remain a barrier [7].

Before COVID-19, telerehabilitation faced various restrictions, but the pandemic led to regulatory changes that accelerated its adoption [8]. During the health crisis, telerehabilitation ensured continuity of care and became an alternative to direct rehabilitation for chronic conditions [9].

Despite efforts to promote digital technology in healthcare, many institutions are not fully utilizing it. Evaluating HCPs' intentions and acceptance of PTR is crucial for successful implementation. The Tele Pulmonary Rehabilitation Acceptance Scale (TPRAS), developed in 2019 by Almojaibel et al., assesses telerehabilitation acceptance among healthcare providers and patients [10]. This scale has demonstrated strong validity and is used to evaluate tele-rehabilitation acceptance in pulmonary programs. Almojaibel et al., in their 2020 study using TPRAS, found that perceived usefulness and ease of use substantially influenced practitioners' intentions to adopt telerehabilitation, with 79% showing positive intentions despite some limitations [11].

While many studies have explored PTR acceptance, there remains a deficiency in comprehending the factors that affect HCPs’ intentions in Saudi Arabia. This study seeks to fill this vacuum by investigating the acceptance of PTR among HCPs in Saudi Arabia, considering the unique cultural and healthcare context.

## Materials and methods

This was a cross-sectional, questionnaire-based, online study, aimed at assessing the acceptance of PTR by HCPs in Saudi Arabia and identifying factors influencing their intention to use it. The study was approved by the Institutional Review Board, Imam Abdulrahman Bin Faisal University, Dammam, Saudi Arabia (approval number: IRB-UGS-2025-03-0038). All participants were HCPs who voluntarily agreed to take part in the study. Informed consent was obtained from each participant at the beginning of the survey questionnaire. Participants were assured of the confidentiality of their responses, and their data were anonymized and used solely for the purposes of this research.

Eligibility criteria

HCPs with experience in PR were recruited from across Saudi Arabia. The sample size, calculated using the online Raosoft sample size calculator (Raosoft Inc., Seattle, Washington, United States), targeted 200 subjects with a margin of error of 6.988% and a 95% confidence level. Table 1 outlines the inclusion and exclusion criteria for study participants.

**Table 1:** Inclusion and exclusion criteria HCP: healthcare practitioner

Inclusion criteria	Exclusion criteria
HCPs in Saudi Arabia who were willing to participate, and had experience in rehabilitation	Individuals currently undergoing healthcare training (e.g., medical interns, students) who have not yet entered independent clinical practice. HCPs whose primary practice is located outside Saudi Arabia, which limits relevance to the study’s geographic context. Participants who declined informed consent. HCPs whose roles do not involve direct rehabilitation care (e.g., administrative or non-clinical positions)

Sampling method

Participants were recruited using a snowball sampling method, an online chain referral technique where initial respondents refer others to participate. The survey was distributed via diverse social media platforms (e.g., WhatsApp (Meta Platforms, Inc., Menlo Park, California, United States), Telegram (Telegram FZ-LLC, Dubai, United arab Emirates), X (Bastrop, Texas, United States), and LinkedIn (LinkedIn Corporation, Sunnyvale, California, United States)), and participants were encouraged to share the survey link within their networks.

Data collection tool and procedure

Data was collected through an online platform (QuestionPro Survey Software; QuestionPro Inc., Austin, Texas, United States) using the pre-validated Tele Pulmonary Rehabilitation Acceptance Scale (TPRAS), developed by Almojaibel et al. in 2019 [10]. The TPRAS consists of two subscales representing the independent variables: perceived usefulness (PU) and perceived ease of use (PEOU). In this study, PU denotes the participants' conviction that the PTR system would provide clinical and practical advantages, while PEOU signifies the opinion that the system could be utilized without much difficulty. Behavioral intention (BI), the dependent variable, which is also a part of the TPRAS, assesses a user's preparedness to utilize PTR in the future. Permission has been taken from the author of the TPRAS via email for its use in this study.

Before completing the questionnaire, participants were informed of the study's objective, anticipated duration, and data confidentiality. Participation was optional, and people might disengage at any moment without repercussions. The survey comprised six sections: (i) Demographic information: age, gender, region, nationality, and occupation; (ii) Work environment details: hospital type, overall years of experience, years of experience in rehabilitation, and years of experience in tele-rehabilitation; (iii) PU: nine questions; (iv) PEOU: four questions; (v) BI: four questions; (iv) potential obstacles: questions concerning the possible obstacles to the implementation of PTR. Responses from sections 3 to 5 were collected using a five-point Likert scale, with values ranging from 1 (strongly disagree) to 5 (strongly agree).

The questionnaire is given in the Appendices.

Statistical analysis

**Figure 1:**
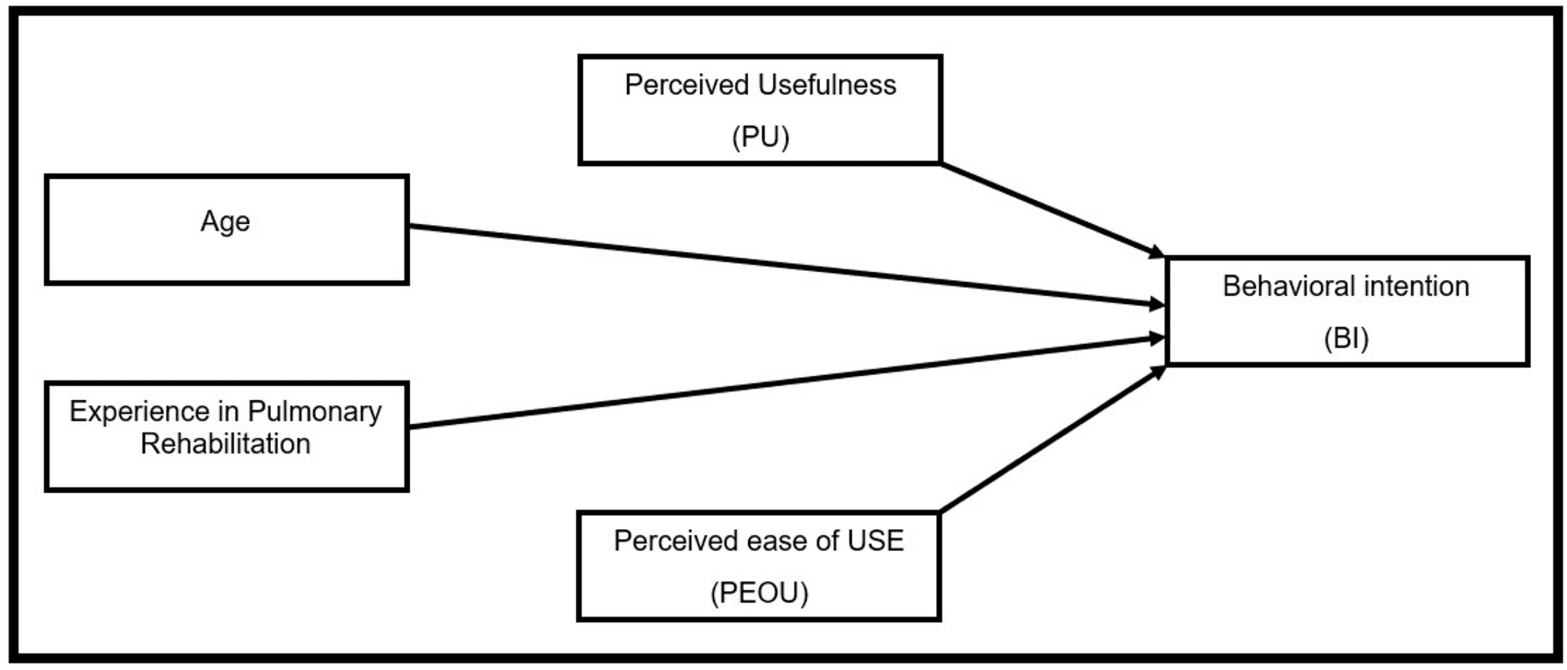
A model predicting participants’ intention to use pulmonary telerehabilitation.

The statistical analysis was conducted utilizing SPSS Statistics for Windows, version 15 (Released 2006; SPSS Inc., Chicago, Illinois, United States). The variables were analysed to obtain the mean and standard deviation. Variables such as gender, region, profession, total years of experience, and barriers were displayed using either bar charts or pie charts. Linear regression was performed on the variables, including independent variables such as PU, PEOU, age, and total years of experience in rehabilitation. The dependent variable was BI. Data was analysed to obtain the statistical significance level (p-value< 0.001) for the model's independent variables and their relationships to the dependent variable (Figure 1).

## Results

A total of 59 HCPs completed the online survey between February 2025 and April 2025. The participants’ ages ranged from 23 to 58 years (mean ± SD: 31.32 ± 6.80). Of the participants, 54 (91.53%) were Saudi, while five (8.47%) were non-Saudi HCPs. The gender distribution showed that the majority were male, with 35 male participants (59.32%) and 24 female participants (40.68%). Most participants worked in the Eastern region (n=27, 45.76%) and the Riyadh region (n=23, 38.98%). Fewer participants were from the Madinah (n=4, 6.78%), Makkah (n=2, 3.39%), Al-Qassim (n=2, 3.39%), and Al-Baha (n=1,1.69%) regions. The majority of participants were respiratory therapists (n=23, 38.98%) and physiotherapists (n=21, 35.59%), and the minority of the respondents were nurses (n=1, 1.69%), nutritionists (n=2, 3.39%), pharmacists (n=2, 3.39%), and physicians (n=2, 3.39%); most of the “others” were occupational therapists (n=8, 13.56%).

**Figure 2:**
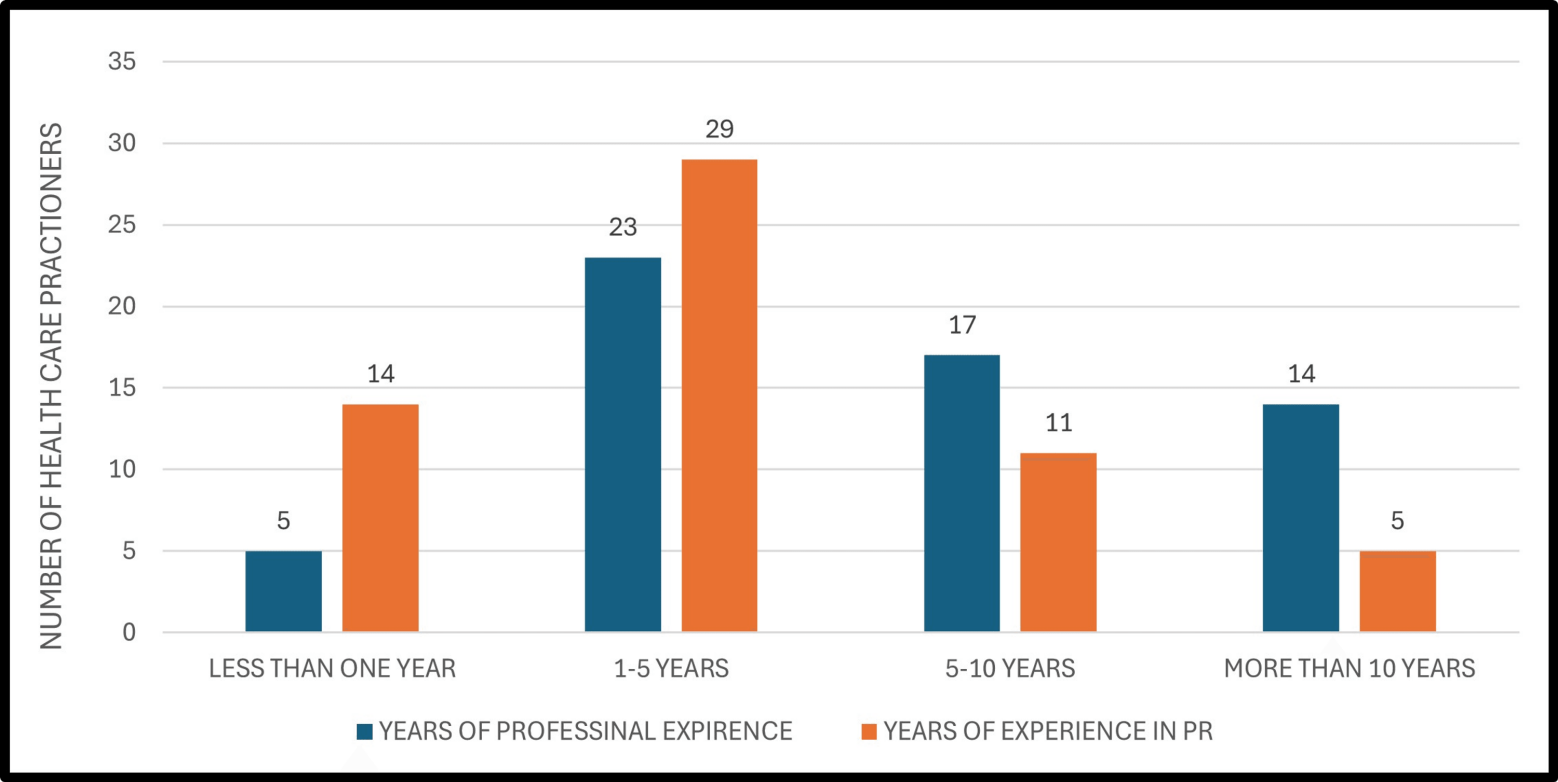
Professional vs. pulmonary rehabilitation experience of healthcare professionals PR: pulmonary rehabilitation

Regarding work settings, most HCPs were employed in private hospitals (n=33, 55.93%), followed by government hospitals (n=10, 16.95%). In terms of experience, a considerable proportion of participants reported having one to five years of general professional experience (n=23, 38.98%) and one to five years of pulmonary rehabilitation experience (n=29, 49.15%). The mean total years of professional experience among all HCPs was 2.68 ± 0.94 years, while the mean years of experience specifically in pulmonary rehabilitation were 3.12 ± 0.87 years. Despite this level of professional experience, many participants (n=36, 61.02%) reported that they had never previously used telerehabilitation. Figure 2 presents a comparative analysis of the HCPs’ overall professional experience and their pulmonary rehabilitation-specific experience.

Perceived usefulness of PTR

**Figure 3:**
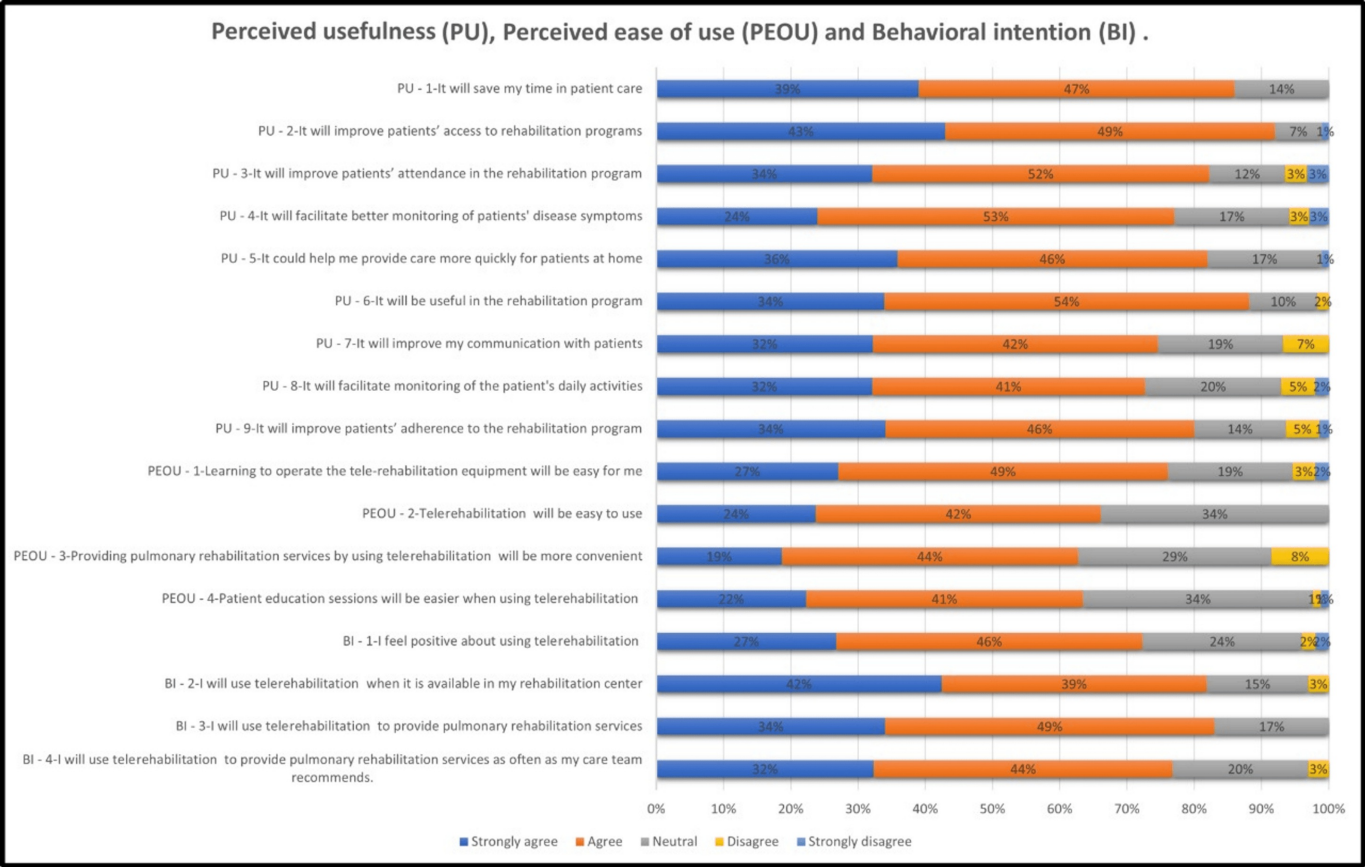
. Responses to the questionnaire on perceived usefulness, perceived ease of use, and behavioral intention on a five-point Likert scale.

The study shows an elevated level of PU of PTR among HCPs. A total of 51 HCPs (86%) accepted that PTR will save time in patient care. Additionally, 91.52% (n=54) positively agreed that telerehabilitation will improve patients' access to rehabilitation programs, and 84.74% (n=51) concurred that it will be beneficial for rehabilitation programs, enhancing patient attendance and adherence to these programs. Furthermore, 72.88% (n=44) agreed that it will improve their communication with patients and facilitate monitoring their daily activities (Figure 3).

Perceived ease of use of PTR

Forty-five HCPs (76.27%) accepted that learning how to operate telerehabilitation equipment will be easy for them, 66.1% (n=39) concurred that telerehabilitation will be easy to use, 62.71% (n=37) believed that rehabilitation services by using telerehabilitation will be more convenient, and an average of 62.71% (n=35) accepted that patient education sessions will be easier and PR services will be more convenient (Figure 3).

Behavioral intention to use PTR

Of the HCPs, 73% (n=43) accepted that they felt positive about using telerehabilitation, and 81.35% (n=48) of HCPs concurred that they will use telerehabilitation when it is available (Figure 3).

Barriers to PTR

**Figure 4:**
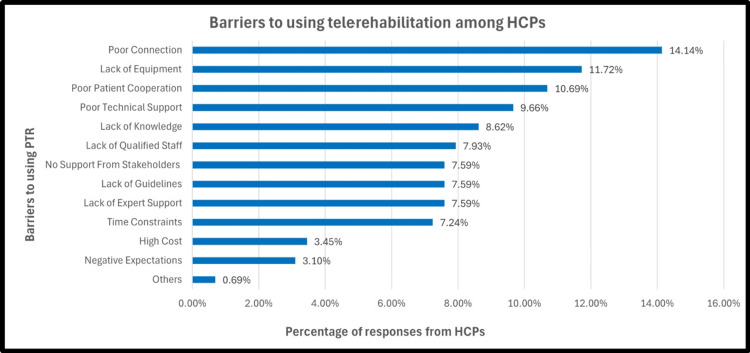
Barriers to using PTR among HCPs PTR: pulmonary telerehabilitation; HCP: healthcare professionals

Regarding potential barriers to the use of telerehabilitation, the most reported issue was poor internet connection (14.14%), followed by poor patient cooperation in using the system (11.72%). Other barriers, such as excessive costs (3.45%) and negative expectations of results (3.10%), were reported less frequently (Figure 4).

Linear regressions were computed for each important variable stated in the study. Specifically, PU, PEOU, rehabilitation experience, and HCP age were evaluated to ascertain their possible beneficial impact on BI to utilize PTR in the future. The analysis indicated that PU significantly positively influenced BI (β = 0.592, t = 5.901, p < 0.001), indicating that HCPs who perceived PTR as useful were more likely to intend to use it. PEOU showed a substantial favorable influence on BI (β = 0.282, t = 2.780, p = 0.007) regarding HCPs’ intention to utilize PTR. Nonetheless, age (β = 0.058, t = 0.539, p = 0.592) and years of rehabilitation experience (β = 0.074, t = 0.687, p = 0.495) were not significant predictors of HCPs’ BI to utilize PTR. The results demonstrate that PU was the most significant predictor of HCPs’ inclination to utilize PTR (Table 5).

**Table 2:** Regression analysis results showing how PU, PEOU, age, and experience in rehabilitation influenced BI

Model	p value	95% CI for BI	
		Lower	Upper
Perceived Usefulness (PU)	< .001>	.467	.947
Perceived Ease of Use (PEOU)	.007	.078	.480
Age	.592	-.016	.027
Experience in Pulmonary Rehabilitation	.495	-.110	.224
Dependent Variable: Behavioral Intention (BI)			

## Discussion

The study found that the level of acceptance of PTR among HCPs in Saudi Arabia was 82.77%. Respondents were aware of its benefits in terms of time saving for themselves and for patients, providing service access, enhancing both participation and compliance when coming to rehabilitation programs, and supporting the monitoring of symptoms. PTR was also found to be easy to learn and use by HCPs, and most of them expressed their readiness and willingness to implement PTR in Saudi Arabia. They also had a positive attitude toward its implementation in clinical practice. However, a few barriers were found, and the main reported barriers were poor internet connection and poor patient cooperation, highlighting the importance of addressing these issues for successful implementation.

Our study's findings, which revealed elevated levels of acceptance of telerehabilitation, align with previous research. Moecke et al. highlighted that telehealth allowed HCPs to attend to more patients in a shorter time, benefiting both providers and recipients [12]. Similarly, Almojaibel et al. found that HCPs were open to adopting and integrating telerehabilitation techniques [10]. Also, Schweiberger et al. observed improvements in access to care [13], and Alghamdi et al. found that patient monitoring was easier and simpler via telehealth, and access to rehabilitation services was less difficult for patients [14].

Inskip et al. identified four essential components that contributed to the success of PTR from both patient and HCP perspectives: social interaction, involvement of HCPs, the ability to track health progress (biometrics), and continued support [15]. Their findings reflected the same positive attitudes and interest shown in the current study, emphasizing that both patients and HCPs appreciated programs that retained the core elements of traditional rehabilitation.

The findings suggest a relationship between PEOU and BI. Bairapareddy et al. reported that 71% of HCPs were aware of using smartphone-based telerehabilitation [7]. Similarly, Fleddermann et al. found that 41% of respondents believed that the equipment would simplify patient education sessions, with 49% considering it easy to use [16]. However, according to Almojaibel et al., no significant link existed between BI and PEOU (p > .05) due to variations in the sample population or environmental factors [11].

The barriers identified in this study also mirrored those found in international research. Giesbrecht et al. reported that technological access posed challenges in Canada and the Netherlands [17], while Moecke et al. emphasized that both technical skill deficiencies and access limitations were significant obstacles [12]. Damhus et al. highlighted inadequate staff training as a key barrier [18], whereas Alghamdi et al. identified time constraints and heavy workloads as major concerns in Saudi Arabia [14]. These findings underscore the importance of enhancing adequate technical training among HCPs.

Brighton et al. conducted a study that involved co-designing a PTR program with both patients and HCPs [19]. Their findings revealed a strong preference among the participants for group-based sessions, while an in-person evaluation before starting telerehabilitation was deemed crucial for ensuring patient safety. Likewise, Verweel et al. explored experiences during the COVID-19 pandemic, identifying key themes such as adapting to the “new normal” and expanding care beyond traditional boundaries, perspectives that closely aligned with HCPs’ views in the current study [20].

The importance of PTR in managing chronic pulmonary conditions such as COPD, especially in underserved communities, has been well documented in the literature despite existing challenges. Selzler et al. established that telehealth rehabilitation can enhance exercise capacity and quality of life to levels similar to conventional approaches [21]. Their findings reinforced structured nationwide initiatives like “Living Well with COPD,” which emphasized patient self-management and system accessibility.

Thus, this study has shown that PTR is accepted in Saudi Arabia. However, overcoming patient and technological obstacles is necessary for the successful implementation and adaptation of PTR. Investing in infrastructure, HCP training, and standardized protocols seems essential for incorporating PTR into clinical practice effectively.

Limitations

One key limitation of this study was the low response rate, with most participants coming from two specific regions, while other regions were underrepresented due to the online chain survey method, which limited outreach. Additionally, the majority of responses were from physiotherapists and respiratory therapists, with minimal input from other healthcare specialties. Despite efforts to encourage wider distribution, limited sharing of the survey by the recipients restricted the sample size.

## Conclusions

Telerehabilitation, particularly PTR, has significantly improved access to rehabilitation services and enabled more efficient patient monitoring. This study examined factors influencing HCPs' acceptance of PTR to evaluate variables such as PU, PEOU, and BI. PU emerged as the strongest predictor of intention to use PTR, with most participants viewing PTR as timesaving, effective for patient monitoring, and easy to learn. Based on these findings, future research should include both patients and HCPs to gain a fuller understanding of PTR acceptance. Additionally, incorporating direct observational methods could provide deeper insights and enhance the validity of future studies.

## Appendices

Survey questionnaire

Consent/Confirmation

Q: Do you consent to participate in this study and allow us to use the data for future publication? Yes/No

Q: Are you a healthcare practitioner? Yes/No

Section 1: Demographic Information

Q: What is your gender? 

Male  

Female 

Q: How old are you? ________ 

Q: Where do you work? 

Riyadh Region / Eastern Region / Mecca Region / Asir Region / Jazan Region / Medina Region / Al-Qassim Region / Tabuk Region / Ha’il Region / Najran Region / Al-Jawf Region / Al-bahah Region / Northern Region 

Q: What is your nationality? 

Saudi 

Non-Saudi 

Q: What is your profession? 

Physician / Nurse / Pharmacist / Respiratory Therapist / Physiotherapist / Nutritionist / Psychologist / Social Worker / Other (please specify): ____ 

Section 2: Work Setting

Q: What type of hospital do you work in? 

Private hospital / Military hospital / University hospital / Primary care / Government hospital / Tertiary hospital / Other (please specify): __________ 

Q: What are your total years of professional experience? 

Less than one year / 1-5 years / 5-10 years / More than 10 years 

Q: What is the total number of years of your experience in pulmonary rehabilitation? 

 I have never worked in the field of Rehabilitation / Less than one year / 1-5 years / 5-10 years / More than 10 years 

Q: What are your total years of experience in tele-rehabilitation? 

I never used telerehabilitation / 1 year or less / 2-4 years / 5 years or more 

Section 3: Perceived Usefulness (PU)

**Table 3:** Questionnaire Section 3: Perceived usefulness of pulmonary telerehabilitation

	Strongly agree	Agree	Neutral	Disagree	Strongly disagree
It will save my time in patient care	❏	❏	❏	❏	❏
It will improve patients’ access to rehabilitation programs	❏	❏	❏	❏	❏
It will improve patients’ attendance in the rehabilitation program	❏	❏	❏	❏	❏
It will facilitate better monitoring of patients' disease symptoms	❏	❏	❏	❏	❏
It could help me provide care more quickly for patients at home	❏	❏	❏	❏	❏
It will be useful in the rehabilitation program	❏	❏	❏	❏	❏
It will improve my communication with patients	❏	❏	❏	❏	❏
It will facilitate monitoring of the patient's daily activities	❏	❏	❏	❏	❏
It will improve patients’ adherence to the rehabilitation program	❏	❏	❏	❏	❏

Section 4: Perceived Ease of Use (PEOU)

**Table 4:** Questionnaire Section 4: Perceived ease of use of pulmonary telerehabilitation

	Strongly agree	Agree	Neutral	Disagree	Strongly disagree
Learning to operate the tele-rehabilitation equipment will be easy for me	❏	❏	❏	❏	❏
Tele-rehabilitation will be easy to use	❏	❏	❏	❏	❏
Providing pulmonary rehabilitation services by using tele-rehabilitation will be more convenient	❏	❏	❏	❏	❏
Patient education sessions will be easier when using tele-rehabilitation	❏	❏	❏	❏	❏

Section 5: Behavioral Intention

**Table 5:** Questionnaire Section 5: Behavioral intention to use pulmonary telerehabilitation

	Strongly agree	Agree	Neutral	Disagree	Strongly disagree
I feel positive about using tele-rehabilitation	❏	❏	❏	❏	❏
I will use tele-rehabilitation when it is available in my rehabilitation center	❏	❏	❏	❏	❏
I will use tele-rehabilitation to provide pulmonary rehabilitation services	❏	❏	❏	❏	❏
I will use tele-rehabilitation to provide pulmonary rehabilitation services as often as my care team recommends.	❏	❏	❏	❏	❏

Section 6: Potential Obstacles

Q: What do you think are the potential barriers to using pulmonary tele-rehabilitation? 

Time constraints or busy schedule / Poor internet connection / Lack of knowledge about the benefits of pulmonary telerehabilitation / Lack of qualified staff / Lack of expert support / Poor technical support / Lack of equipment to improve the effectiveness of pulmonary telerehabilitation / Poor patient cooperation in using pulmonary telerehabilitation / Lack of laws, regulations, and guidelines for implementation / No support from stakeholders (like employees, investors, etc.) / High cost / Negative expectations of results/ Others: Please mention 

Q: Do you have any recommendations? ______________________________________
